# Modeling Time to Cure of Deep Vein Thrombosis Using Cox Proportional Model in Southwest of Ethiopia

**DOI:** 10.4314/ejhs.v32i3.11

**Published:** 2022-05

**Authors:** Gurmessa Nugussu Gelcho, Mosisa Girma Bekele

**Affiliations:** 1 Department of Statistics, College of Natural Sciences, Jimma University, Jimma, Oromia, Ethiopia

**Keywords:** Deep vein thrombosis, Cox proportional hazard, Cardiovascular

## Abstract

**Background:**

Globally, there are about 10 million cases of deep vein thrombosis every year, and it is the third leading cardiovascular disease after myocardial infarction and stroke. The objective of the study is to assess risk factors of time to cure patients of deep vein thrombosis in southwest Ethiopia.

**Methods:**

A retrospective cohort study design was used. The study population was deep vein thrombosis patients at purposively selected hospitals in Southwest Ethiopia from January 2017 to December 2020. Cox proportional hazard model was used to identify risk factors associated with deep vein thrombosis.

**Results:**

Out of the total 1068 registered as deep vein thrombosis patients, 263(24.6%) were cured during the study period, and 805(75.4%) were censored. Results of the Cox proportional hazard model show that; age, gender, family history of deep vein thrombosis, smoking status, immobilize and alcohol consumption were factors associated with deep vein thrombosis (p-value<0.05).

**Conclusion:**

The patients with a family history of deep vein thrombosis, prolonged immobilization, greater the 50 years, smoking cigarettes, female (non-pregnant) and alcohol users had a longer curing time of deep vein thrombosis compared to others.

## Introduction

Venous thromboembolism (VTE) is the formation of a blood clot in a deep vein that can lead to complications including deep vein thrombus (DVT) and pulmonary embolism (PE). Venous thromboembolism is a serious condition with an incidence of 10% to 30% of people dying within one month of diagnosis and half of those diagnosed with a DVT have long-term complications (1). Deep vein thrombosis (DVT) is the development of single or multiple blood clots within the deep veins of the extremities or pelvis, usually accompanied by inflammation of the vessel wall (2). DVT is the third most common cardiovascular disease after myocardial infarction and stroke, and it is a growing public health problem due to an increase in the aging population (3). The development of single or multiple blood clots within the deep veins of the extremities or pelvis, usually accompanied by inflammation of the vessel wall and which is common affects the leg veins (such as the femoral vein or the popliteal vein) or the deep veins of the pelvis which made them common sources of serious complications (1).

Approximately 10 million cases of DVT occur every year across low, middle, and high-income countries worldwide93). Over 2 million people develop DVT in the USA alone every year (4). In Europe, more than 500,000 deaths occur annually as a complication of deep vein thrombosis (5). In England, an estimated 25,000 a year die from hospital-related VTE.

In Africa, the prevalence of DVT varies between 2.4% and 9.6% according to the different studies in African countries (6). The prevalence of deep vein thrombosis in the population of South Africa is 0.10% a year (7). In Nigeria, the prevalence of DVT is 2.9%, with increased risk in male patients older than 40 years and in those with active cancer (8).

Risk factors of DVT are multifactorial and can be classified as modifiable or non-modifiable. Modifiable risk factors include immobility, HIV infection, sepsis, malignancy, heart failure, renal failure, diabetes mellitus, obesity, long travel, trauma, and surgery (6–9). Non-modifiable risks include gender, age, race, and hereditary risk factors such as antithrombin, Prothrombin gene mutation, protein S, and protein C deficiency (9–10).

Deep venous thrombosis (DVT) is a common clinical problem associated with substantial morbidity and mortality (11). Deep vein thrombosis (DVT) disease was estimated to account for 1 in 4 deaths worldwide in 2017 and increases the cause of mortality. In developed countries, deep vein thrombosis is a serious public health problem and ranks among the main causes of mortality (12).

Complications from deep vein thrombosis can be very serious. They can include pulmonary embolism (PE), chronic venous insufficiency, and post-thrombotic syndrome. The prospective study done in Ethiopia revealed that the DVT recurrence rate was high, which is even complicated with pulmonary embolism as well as death (11).

Mortality from DVT cases is also increasing in non-developed countries including Ethiopia. Knowledge of the global burden of DVT recurrence is deficient in Africa, including Ethiopia (11). However, to our knowledge, there is a lack of study conducted in Ethiopia on survival analysis of factors associated with deep vein thrombosis patients in Ethiopia, especially in the Southwest of the country.

## Methods

**Study area and source of data**: The study was conducted at five hospitals selected purposively, namely; Jimma University Medical Center, Bedelle, Metu-Karl, Mizan Amin, and Bonga hospital starting from January 2017 to December 2020 in Southwest Ethiopia.

The required data for this study were extracted from follow-up charts and cards of DVT patients admitted to the purposively selected hospital from January 2017 to December 2020. Data for this research were collected by healthcare professionals under the supervision of statisticians.

**Study population and sampling techniques**: The study population was all DVT patients who had been registered at five hospitals purposively selected in Southwest of Ethiopia. In total, 1068 patients with DVT from the five hospitals were included in the study. The response variable was time to cure deep vein thrombosis disease (in the day). The explanatory variables were gender, residence, age, alcohol consumption, khat chewing and smoking behavior active cancer, prior history of surgery, obesity, immobilization, history of trauma, and history of air travel.

**Study design**: A retrospective study design was employed and the data were obtained from a cohort of DVT patients admitted to the five hospitals purposively selected in Southwest of Ethiopia. The study period was from January 2017 to December 2020, with a 24 hour's follow-up period. The data was entered by using Epi Info (version 7). Entry of the data was considered from the date of initial registration as DVT patients until the patients were discharged from the hospital with less than two (2) international normalized ratios (INR). The international normalized ratio is the lab result and calculated based on the results of a prothrombin time. The time was measured by days in this study, and the data were analyzed using R software (version 4.1.2).

**Inclusion and exclusion criteria**: This study included all DVT patients registered from January 2017 to December 2020 who received DVT treatments or continued the follow-up except pregnant women; they have a high probability to develop DVT. This is may be pregnant women are more likely to coagulate, due to excessive bleeding during pregnancy, miscarriage, and childbirth. On the other hand, unregistered patients' outcome or those who have no full information about variables of interest was also excluded from the study.

**Statistical methods**: The study focused on time to event (time to cure of DVT), so the appropriate method of this particular study is survival analysis. We have performed the Kaplan-Meir estimator and Cox proportional hazard model for the analysis and model building. We have also used log-rank tests and Wilcoxon tests for comparison of survival functions. Kaplan Meier analysis was used to study survival patterns; the KM plot, which is a step function, gives some indications about the shape of the survival distribution. The figure in general shows if the pattern of one survivorship function lies above another which means the group defied by the upper curve lived longer or had a more favorable survival experience than the group defied by the lower curve.

Proportional Hazard Model is used for multivariate analysis to identify factors associated with death from tuberculosis and Cox proportional hazards (PH) model given by

*λ*(*t|Z*) = *λ*_0_(*t*)*e^ZTβ^*,

Where, *Z* = (*Z*_1_, *Z*_2_..., *Z_p_*)^*T*^ and *β* = *β*_1_, *β*_2_, ..., *β_p_*)^*T*^**Z** is px1vector of covariates such as treatment indicators and prognostic factors, and *β* is a × 1 vector of regression coefficients. The parameter was estimated by using partial likelihood functions. We used the partial likelihood ratio test to assess the significance of the coefficients in the Cox proportional hazards model.

**Ethics approval and consent to participate**: Ethical approval was obtained from the Institutional Research Ethics Review Committee of the Jimma University College of Natural Sciences. A letter of support was written for the five-hospital purposively selected among hospitals in Southwest Ethiopia. The secondary data were collected without including identities (e.g., names) of the patients by professional healthcare.

## Results

**Descriptive summaries of characteristics of patients**: Of all 1067 Deep vein thrombosis patients, 263(24.65%) were cured (Table 1). According to the result, the prevalence of non-pregnant women was 46.5 %, and men (28.9%) were censored, whereas 13% of non-pregnant women and 11.6 % of men were cured. The median cure time of non-pregnant women and men was 16 days and 13 days respectively. Out of the entire subjects integrated into this study, 9.8% of patients who lived in urban were cured and 14.8% in rural were cured. The median cure time of urban and rural was 19 days and 18 days respectively. The median cure time of patients who have a previous history and who have no previous history of DVT was 23 and 18 days respectively. The study revealed that 5.7% of patients who had a previous history of DVT were cured and 18.9% of patients who had no previous history of DVT were cured. Also, the table provided information about the status of smoking. According to the results, among 122 (11.43%) patients who smoked, 3.7% were cured of DVT while (88.57%) patients who had not smoked, 23.7% were cured.

**Table 1:** Descriptive summary of the covariates associated with DVT patients

Covariates	censored		cured		Median time (in days)
	Number	Percent	Number	Percent	(95%CI)
Gender					
Female	496	46.5	139	13.0	16(15,18)
Male	308	28.9	124	11.6	13(11,14)
Residence					
Urban	309	29	105	9.8	19(15,21)
Rural	495	46.4	158	14.8	18(17,22)
Age of patients					
≤ 35 years	117	11.0	180	16.9	18(16,20)
>35 years	687	64.4	83	7.8	15(13,16)
Previous history of DVT					
Yes	124	11.6	61	5.7	23(20,28)
No	680	63.7	202	18.9	18(15,21)
Family history of DVT					
Yes	37	3.5	65	6.1	20(19,22)
No	767	71.9	198	18.6	18(14,19)
Smoking status					
Yes	83	7.8	39	3.7	19(17,21)
No	721	67.6	224	21.0	18(15,21)
Immobilize					
Yes	79	7.4	47	4.4	13(11,16)
No	725	67.9	216	20.2	10(9,13)
Alcohol consumption					
Non-alcohol user	585	54.8	103	9.7	9(8,12)
Alcohol user	219	20.5	160	15.0	13(10,16)

In this study, it was found that 54.8% were non-alcohol users. It was observed that among non- alcohol user patients, 9.7% were cured of DVT, while 15% of the patients who were alcohol users were cured. This indicates that non-alcohol user patients were more cured than alcohol user patients. The median cured time of non-alcohol user patients was 9 days which is less than alcohol user patients (13 days).

**Result of Cox proportional hazard model:** Cox proportional hazard model was used to find the factors associated with DVT patients in Southwest of Ethiopia. In Table 2, the covariates associated with deep vein thrombosis were analyzed by using the cox proportional hazard model. Accordingly, the estimated hazard ratio (HR) of male patients is 1.292(95% CI: 1.008, 1.654) implying that the probability of cured men is 12.92% higher than non-pregnant women (reference group) controlling for other covariates in the model (Table 2). The confidence interval indicates that the cured men are as low as 1.008 and as high as 1.654 times the probability of cure of non-pregnant women.

**Table 2:** Multivariate analysis of Cox proportional hazards model of DVT patients in Southwest Ethiopia

Covariates	Estimate	SE	P-value	HR (95% CI)
Gender				
Male	0.256	0.126	0.043*	1.292(1.008,1.654)
Female	Ref.			
Age of patients				
≤50 years	0.432	0.138	0.032*	1.641(0.541,2.147)
>50 years	Ref.			
Family history of DVT				
No	0.896	0.149	<0.001*	2.449(1.829,3.279)
Yes	Ref.			
Smoking status				
No	0.424	0.179	0.018*	1.528(1.076,2.168)
Yes	Ref.			
Immobilize				
No	0.628	0.166	<0.001*	1.873(1.352,2.596)
Yes	Ref.			
Alcohol consumption				
Alcohol user	-0.585	0.129	<0.001*	0.555(0.431,0.715)
Non-alcohol user	Ref.			

* Significant at 5%

The estimated hazard ratio (HR) of the patients from a rural area is 2.055(95% CI: 1.602, 2.636) implying that the probability of cured rural patients is 20.55% higher than urban (reference group) controlling for other covariates in the model. The confidence interval indicates that the cured rural patients are as low as 1.008 and as high as 1.654 times the probability of cure for urban patients.

The estimated HR of the patients who had below 35 years old is 2.205 (95%CI 1.715, 2.835). A patient who had below 35 years old has a 20.5 times higher chance of cure than a patient who had above 35 years old (reference group) controlling for other covariates. The confidence interval implies that the chance of cure for patients who had below 35 years old is as low as 1.715 (71.5%) and as high as 2.835 times the chance of cured patients who had above 35 years old.

The estimated HR for covariate for family history of DVT is 2.449(95%CI: 1.829, 3.279). This implies that the patients who had no family history of DVT had 44.9% higher cured than patients who had a family history of DVT (reference group) controlling for other covariates in the model. The confidence interval suggests that the hazard rate for patients who had no family history of DVT is as low as 1.829(82.9%) and as high as 3.279 (27.9%) times cured for patients who family had history of DVT.

The estimated HR for patients who did not mobilize is 2.055(95%CI: 1.602, 2.636). This shows that a patient who was no mobilized had a 5.5% longer time of cure than patients who were mobilized (reference group) controlling for other covariates. The confidence interval indicates that the chance of a patient who was not mobilized being cured is as low as 1.602 (60.2%) and as high as 2.636 (63.6%) times the chance of a patient who was mobilized being cured. The study clarified that the hazard ratio for the alcohol user patients was 0.555(95% CI 0.431, 0.715).

The estimated times of cure for the alcohol user patients was 55.5 times higher as compared to non-alcohol users (reference group) controlling for other covariates. The confidence interval indicates that the cured time for the alcohol user patients was as low as 0.431 (43.1%) and as high as 0.715 (71.5%) times the chance of cure than patients who were non-alcohol users.

## Discussion

The study revealed that men had a shorter cure time than non-pregnant women patients. The finding was consistent with those of other studies conducted in Sudan (13). This similarity may be due to blood in women being more likely to coagulate than that in men because this property of blood in women protects against excessive bleeding during pregnancy, miscarriage, and childbirth.

The finding of this study revealed that the age of the patients had an effect on the time to cure DVT. The result of this study was in line with those of other studies performed in Ethiopia (11), and in Canada (14) which found that the risk of DVT increases with age. This similarity may be due to as age increases, so does the risk. This increase in risk appears to be related to several age-related factors, mobility, an increased number of other major risk factors such as cancer, age-related changes in the bloods tendency to clot that contribute to the development of DVT, and induce a greater risk of generating VTE.

In this study, it was found that patients who were non-alcohol users had a shorter curing time than alcohol users. This finding was in line with previous studies conducted in Ethiopia (11), in USA (15), in Norway (16) which revealed that the frequent binge drinkers (≥ 1/week) had a 17% increased risk of DVT. This could be due to drinking too much damaging the liver, where alcohol is processed, when the liver becomes damaged, it reduces the ability to produce proteins that regulate blood clotting which increases blood clotting.

In this study, patients whose families had no history of DVT had a shorter curing time than those who had a previous history of DVT. The finding was consistent with the previous studies conducted in Ethiopia (11), family history was a risk factor for recurrent DVT in US (15), genetics can play a role in the development of DVT in Sudan (13), and family history of DVT is a modest risk factor for recurrence of DVT in Sweden (17).

Similarly, the finding of this study showed that prolonged immobilization had an effect on the curing time of DVT patients. The study was consistent with the previous studies done in Ethiopia (11), in Sudan (13), in USA (15), in Canada (14), and in Sweden (17). This may be due to bed rest or other immobilization pooling blood in the veins for long periods of time which is associated with an increased risk of DVT. Immobilization is also associated with other medical conditions such as hemiplegia due to a stroke which leads to local venous stasis by the accumulation of clotting factors and fibrin, resulting in blood clot formation.

Also, this study found that the smoking status of the patients had an effect on cure time from DVT. The results of this study were in line with the previous studies performed in Ethiopia (11), in Sudan (13), and in USA (15). This similarity may be due to smoking changing the surface of blood platelets, damaging the lining of blood vessel walls which block the blood vessel and increase the potential for clots to form. When the blood is thinner, the heart is hard to work to move around the body which leads to DVT. The covariates significantly affect the DVT patients were age, gender, family history of deep vein thrombosis, smoking status, immobilize and alcohol consumption of patients. The DVT patients with a family history of DVT, prolonged immobilization, greater the 50 years, smoking cigarettes, female (non-pregnant) and alcohol user had longer cure time of DVT compared to others.
